
JOURNAL INFORMATION
==============================
NLM Title Abbreviation: South Med Rec
NLM Journal ID: 0027317

The Southern Medical Record

Publisher: Biomedical Journal Digitization Project

ARTICLE INFORMATION
==============================
PMCID: PMC9074466
PMID: 36021792
Article version: 1
Subjects: Editorial and Miscellaneous

Pamphlets Received

Print publication date: 1881 Mar 20
Volume: 11
Issue: 3
First page: 120
Last page: 120
License: This work is free of known copyright restrictions. For more information, please see PMC Back Issue Digitization.
License URL: https://creativecommons.org/publicdomain/mark/1.0/

==============================

*Committee of Arrangements.*—Dr. F. D. Cunningham, Richmond, Va., Chairman.

*Amendment to the By-Laws* offered by Dr. J. M. Keller, Ark.: In the election of Officers and the appointment of Committees by this Association and its President, they shall be confined to members and delegates present at the meeting, except in the Committees of Arrangements, Climatology, and Credentials.

W. H. ATKINSON, Permanent Secretary.

---

### *PAMPHLETS RECEIVED.*

ROCKY MOUNTAIN HEALTH RESORTS. AN ANALYTICAL STUDY OF HIGH ALTITUDES IN RELATION TO THE ARREST OF CHRONIC PULMONARY DISEASE. By Charles Denison, A. M., M. D., Reporter to the International Medical Congress, Philadelphia, 1876, on "the influence of high altitudes upon the progress of Phthisis"; Author of "Reports on Climate and Consumption" to the American Medical Association, etc. Second edition.

PHTHISIS PULMONALIS AND ITS TREATMENT WITH HYPOPHOSPHITES. By Le de Bremon, M. D., University of Paris (France), Knight of the Legion of Honor; Member of the New York County Medical Society, the Neurological Society of New York, and the New York Medico-Legal Society; Late Clinical Assistant to Dr. J. F. Churchill, Paris, etc., etc.

A DEVICE TO FACILITATE THE REMOVAL OF DEEP WIRE SUTURES IN THE OPERATION FOR RUPTURED PERINEUM. By Aug. F. Erich, M. D., Professor of the Diseases of Women, College of Physicians and Surgeons, Baltimore, Md.

---

### *RECEIPTED.*

[Receipts not acknowledged privately are entered here.]

1880—Drs. R A McIntosh, B H Mathews, T W Spruell.

1881—Drs. J R Wilson. W H Pool, D S Aswell, T L Doster, J H Blain, E A Anderson, J F Pou, W F Jacobs, M H [illegible]ys, F R Van Eaton, J M Jackson, R J Tolbert, J H Jennings, E Wheeler, T L Quilli[illegible]n, P P Terry, Joseph Underwood, B M Walker, Jno R Mason, W F Clary, J D Jordan, J S Milling, T F Babbitt, T S Parrham, J W Gilbert, A Harris, J M Stansell, C P Sanders, W R Brawner, B T Phipps, W S Glass, H Allison, J B Rumph, J O Sanders, J R Johnson, W P Chapman, Louis Hadden.

---

### SPECIAL NOTICES.

---

**WILLIAM R. WARNER & CO.**, Philadelphia, are furnishing the Profession with pure chemicals and a great variety of Preparations of most reliable and excellent quality. Their SUGAR-COATED PILLS have an established reputation for solubility and purity. Their PARVULES, in the matter of neatness, beauty, variety and convenience of administration, are unsurpassed, and are among the excellences which characterize the advanced condition of drug manufacture of the present age.

**PARKE, DAVIS & CO.**, Detroit, Mich.—The SUGAR-COATED PILLS of the above extensive establishment we have found to be of excellent quality, and are put up in a neat and elegant style. We may say the same of their CAPSULES and GELATINE-COATED PILLS. These beautiful preparations are most convenient for the practitioner, relieving him of the trouble of preparing his medicines and furnishing him everything to hand in a shape suited to every emergency, and most agreeable and acceptable to the patient.

The advertisement of the now celebrated SADDLE BAG, **ELLIOTT'S PATENT**, appears in this issue of our Journal. It is owned and manufactured by A. A. Mellier, of St. Louis, whose house has been established for 25 years, and is among the leading Wholesale Drug establishments of the country. He obtained the patent right of this BAG four years ago, and showing its merits to the United States government, it was adopted and 300 were immediately ordered, and are now in active use; a very clear evidence of superiority.
